# X-ray and UV radiation-damage-induced phasing using synchrotron serial crystallography

**DOI:** 10.1107/S2059798318001535

**Published:** 2018-04-06

**Authors:** Nicolas Foos, Carolin Seuring, Robin Schubert, Anja Burkhardt, Olof Svensson, Alke Meents, Henry N. Chapman, Max H. Nanao

**Affiliations:** a Structural Biology Group, European Synchrotron Radiation Facility, 71 Avenue des Martyrs, 38000 Genoble, France; b Center for Free-Electron Laser Science, Deutsches Elektronensynchrotron, Notkestrasse 85, 22607 Hamburg, Germany; c The Hamburg Centre for Ultrafast Imaging, Luruper Chaussee 149, 22761 Hamburg, Germany; d Integrated Biology Infrastructure Life-Science Facility at the European XFEL (XBI), Holzkoppel 4, 22869 Schenefeld, Germany; e Institute for Biochemistry and Molecular Biology, University of Hamburg, Notkestrasse 85, 22607 Hamburg, Germany; f Photon Science, Deutsches Elektronensynchrotron, Notkestrasse 85, 22607 Hamburg, Germany; g Department of Physics, University of Hamburg, Luruper Chaussee 149, 22761 Hamburg, Germany

**Keywords:** synchrotron serial crystallography, radiation-damage-induced phasing, experimental phasing, radiation damage, genetic algorithms

## Abstract

Specific radiation damage can be used to determine phases *de novo* from macromolecular crystals. This method is known as radiation-damage-induced phasing (RIP). One limitation of the method is that the dose of individual data sets must be minimized, which in turn leads to data sets with low multiplicity. A solution to this problem is to use data from multiple crystals. However, the resulting signal can be degraded by a lack of isomorphism between crystals. Here, it is shown that serial synchrotron crystallography in combination with selective merging of data sets can be used to determine high-quality phases for insulin and thaumatin, and that the increased multiplicity can greatly enhance the success rate of the experiment.

## Abstract 2

Multi-crystal serial crystallography data can be used for UV and X-ray radiation-damage-induced phasing.

## 1. Introduction   

Radiation induces many changes in macromolecular crystals. Amongst these is a reduction in occupancy or the movement of atoms, which is referred to as specific radiation damage. Specific radiation damage can be induced by X-ray or UV light and affects metals, S^γ^ atoms in disulfides, thiol linkages and terminal O atoms in carboxylates (with the latter only being induced by X-rays; Ravelli & McSweeney, 2000 ▸; Burmeister, 2000 ▸; Weik *et al.*, 2000 ▸; Pattison & Davies, 2006 ▸). Specific radiation damage can be of major concern to practitioners of macromolecular crystallography (MX), but in some cases such damage can be used to determine phases experimentally (Ravelli *et al.*, 2003 ▸, 2005 ▸; Zwart *et al.*, 2004 ▸; Banumathi *et al.*, 2004 ▸; Weiss *et al.*, 2004 ▸; Schiltz *et al.*, 2004 ▸; Ramagopal *et al.*, 2005 ▸; de Sanctis & Nanao, 2012 ▸; de Sanctis *et al.*, 2016 ▸). This technique is called radiation-damage-induced phasing (RIP) and, by analogy to single isomorphous replace­ment (SIR), two data sets are used to calculate differences in structure factors (between damaged and less damaged states). However, unlike in SIR, no soaking of heavy atoms is required. If the decrease in occupancy at specific sites is large enough and global radiation damage has been minimized, the positions of radiation damage can be determined. UV RIP generally has the advantage of inducing less general global radiation damage compared with X-ray RIP (Nanao & Ravelli, 2006 ▸; de Sanctis *et al.*, 2016 ▸). When performed on a single crystal or indeed at the same position of single crystals, RIP has the advantage of relatively high isomorphism between the damaged and undamaged data sets. This is a key difference between RIP and traditional isomorphous methods, in which the experiment is performed on different crystals and the introduction of a heavy atom frequently introduces non-isomorphism. Depending on the ratio of specific to global damage, the number of sites and their susceptibility, a wide range of relative changes to intensities can be expected. Initial estimates of the maximal signal based on Crick & Magdoff (1956 ▸) suggested that even modest reductions to occupancies of 26% for six disulfide S atoms could lead to changes in intensity of 10% at 2θ = 0 (Crick & Magdoff, 1956 ▸; Ravelli *et al.*, 2003 ▸). In practice, a wide range of *R* values between damaged and undamaged data sets have been observed: up to 14% overall for trypsin despite low (∼4%) internal *R* values (Nanao *et al.*, 2005 ▸). This differentiates RIP from the other dominant phasing method based on endogenous chemical groups: long-wavelength sulfur SAD. Thus, the potentially high signal and the lack of a requirement for chemical modification of crystals provides a potentially useful alternative method to traditional isomorphous and anomalous methods. However, one key limitation of X-ray and UV RIP approaches is that a minimum of two complete data sets must normally be collected. Two solutions to this limitation are to collect one large data set and subdivide it into two sub-data sets in a ‘segmented RIP’ analysis (de Sanctis & Nanao, 2012 ▸) or to model specific damage as a function of dose, as in *SHARP* (Schiltz *et al.*, 2004 ▸; Schiltz & Bricogne, 2008 ▸, 2010 ▸). In segmented RIP, one collects a large high total dose data set, and the first images collected are treated as a low-damage data set and the last images are treated as a damaged data set. Finally, in cases of large crystals, multiple positions can be collected from a single crystal, allowing the measurement of one complete low-damage data set prior to UV/X-ray exposure. However, the utility of this approach is limited by the trend towards smaller crystals, as well as by intra-crystal non-isomorphism. In UV RIP experiments, the amount of damage depends on the UV source, on the composition of the unit cell and on the crystal volume. In particular, the limited light-penetration depth in macromolecular crystals is a significant challenge to the homogenous illumination of larger crystals. Thus, using small crystals has significant advantages if complete data sets can be collected. While penetration depth is not an issue for X-ray damage, improvements to phasing can be expected if high-multiplicity data sets can be collected. To this end, we have employed recent developments in synchrotron serial crystallography (SSX) to greatly increase the recorded signal at a given dose by combining data from multiple crystals (Diederichs & Wang, 2017 ▸). A major challenge in implementing SSX-RIP is to efficiently deal with non-isomorphism between crystals. Simulated diffraction patterns for free-electron laser serial femtosecond crystallography (SFX), where there is no rotation during exposure, have indicated that such an approach is possible, but it has not yet been demonstrated experimentally (Galli, Son, White *et al.*, 2015 ▸; Galli, Son, Barends *et al.*, 2015 ▸). Here, we show for the first time that SSX can be used to successfully phase macromolecular crystals of thaumatin and insulin *de novo* by X-ray RIP and UV RIP, and explore the relationship between dose, multiplicity and RIP signal.

## 2. Methods   

### 2.1. Crystallization   

The thaumatin crystals used for the X-ray RIP experiment were prepared as described in Nanao *et al.* (2005 ▸). The cubic insulin crystals used for the UV RIP experiment were obtained from porcine insulin purchased from Sigma–Aldrich (catalogue No. I-5523). Crystals of cubic zinc-free insulin were grown *via* hanging-drop vapour diffusion by mixing 4.5 µl protein solution at a concentration of 1.5 mg ml^−1^ in 0.05 *M* sodium phosphate, 0.01 *M* ethylenediaminetetraacetate tri­sodium salt (Na_3_EDTA), pH 10.4–10.8 with 1.5 µl reservoir solution [0.05 *M* sodium phosphate buffer, 0.01 *M* Na_3_EDTA, 20%(*v*/*v*) ethylene glycol pH 10.4]. The mixture was equilibrated against 500 µl reservoir solution. Single crystals of ∼6 × 6 × 6 µm in size were obtained after 1–2 days at 298 K.

### 2.2. Crystal harvesting   

Thaumatin samples were prepared using a buffer with glycerol as a cryoprotectant at a final concentration of 20%, and the crystal slurry of ∼20 × 20 × 20 µm crystals was then harvested with micro-meshes (MicroMeshes with 10 µm holes; MiTeGen catalogue No. M3-L18SP-10). Cubic insulin was directly harvested on silicon chips (Supplementary Fig. S1; Roedig *et al.*, 2016 ▸, 2017 ▸).

### 2.3. Data collection   

Data were collected at 100 K using a Dectris PILATUS3 2M detector on the ID23-2 microfocus beamline (fixed energy 14.2 keV) at the European Synchrotron Radiation Facility (Flot *et al.*, 2010 ▸). UV illumination of insulin crystals was performed using high-power UV-LEDs as described by de Sanctis *et al.* (2016 ▸). Data collection was performed using the *MeshAndCollect* workflow (Zander *et al.*, 2015 ▸). The RIP workflow uses this approach, but performs multiple collections at each position identified from the diffractive map. No explicit X-ray burn was implemented in this workflow, and the data-collection parameters were 100 frames of 0.1° oscillation with 30 ms exposure time at 8.74 × 10^10^ photons s^−1^ and 0.8728 Å wavelength with a beam size of 10 × 8 µm, chosen such that the approximate dose regime would reach 1–4 MGy over the course of six data collections. The dose regime was estimated with *RADDOSE*3*D* based on the crystal dimensions and photon flux (Zeldin *et al.*, 2013 ▸). This particular range was chosen based on previous work, which showed that the RIP signal is optimal at ∼2 MGy (Bourenkov & Popov, 2010 ▸; de Sanctis & Nanao, 2012 ▸). In each successive exposure 100 frames of 0.1° oscillation were collected, resulting in 10° sub-data sets. The same oscillation range was used for each sub-data set. The first exposure was then used as the ‘before’ data set and subsequent exposures as the ‘after’ data set. The ‘before’ data set, while not damage-free, has the lowest dose. The ‘after’ data set is the highest dose, most damaged data set.

### 2.4. Data processing   

Data reduction was performed using *XDS* (Kabsch, 2010 ▸) through the *GreNAdeS* automated pipeline at the ESRF (Monaco *et al.*, 2013 ▸) and data were reprocessed using the REFERENCE_DATASET keyword. All diffraction images have been deposited with Zenodo (https://doi.org/10.5281/zenodo.1035765). Because even in these model systems there can be some variation in data quality and isomorphism between the sub-data sets, selection of only some of the sub-data sets for merging was performed. This was performed using the *CODGAS* genetic algorithm (GA; Zander *et al.*, 2016 ▸). *CODGAS* applies principles of biological natural selection in order to select which sub-data sets to merge, based on a target function which is composed of merging statistics [for example 〈*I*/σ(*I*)〉, *R*_meas_, CC_1/2_ and completeness]. Different potential merging solutions are randomly generated using default target-function weights, followed by rounds of optimization by maximizing the target function.

### 2.5. Substructure determination   

Each pair of data sets (‘before’ and ‘after’) was then treated as a standard RIP experiment, varying the scaling (*K*) of the before and after data sets in *SHELXC*, which offers a native implementation of the RIP phasing strategy, as described in Nanao *et al.* (2005 ▸), Ravelli *et al.* (2005 ▸) and Sheldrick (2010 ▸). Varying the scaling (*K*) and running *SHELXC*/*D*/*E* was performed using a Perl script. The sampling of *K* was from 0.97 to 1.01 in increments of 0.00211. Substructure determination was performed in *SHELXD* using NTRY 5000, SHEL 500 2.2 and FIND 9 for thaumatin, and NTRY 5000, SHEL 500 2.0 and FIND 6 for insulin. The high-resolution limits were chosen based on the resolution at which 〈*d*′/σ(*d*′)〉 drops below 1.5.

### 2.6. Phasing and phase improvement   

Phasing and phase improvement was performed in *SHELXE* using solvent flattening and five cycles of autobuilding (Sheldrick, 2010 ▸; Thorn & Sheldrick, 2013 ▸).

### 2.7. Refinement and *a posteriori* analysis   

*ANODE* (Thorn & Sheldrick, 2011 ▸) was used for the determination of *F*_o_ − *F*_o_ model-phased RIP difference electron-density map peak heights. For both this calculation and the evaluation of phase errors, a refined atomic model was used. The refinement procedure was as follows. Molecular replacement was performed using *MOLREP *(Vagin & Teplyakov, 2010 ▸) with PDB entry 5fgt for thaumatin and PDB entry 9ins for insulin. The models were rebuilt manually in *Coot* and then refined using *BUSTER* (Emsley *et al.*, 2010 ▸; Bricogne *et al.*, 2011 ▸). The final refinement step was performed with the *PDB_REDO* webserver (Joosten *et al.*, 2014 ▸) in both cases. The weighted mean phase errors (wMPE) were calculated using *SHELXE* with the -x option and the same refined model as was used in *ANODE* (Sheldrick, 2010 ▸). The substructure correctness was calculated with *phenix.emma* (with default parameters, except for ‘tolerance’, which was set to 1.5 Å), using a reference pseudo-atom substructure which was generated by *ANODE* with the *F*_A_ data from *SHELXC* in RIP mode (Adams *et al.*, 2010 ▸; Thorn & Sheldrick, 2011 ▸).

## 3. Results   

### 3.1. Data quality   

Each data set acquired from both thaumatin and insulin microcrystals in the *MeshAndCollect* workflow was merged using *CODGAS* to obtain complete data sets. The high-resolution limit was chosen based on the bin with a CC_1/2_ higher than 25% (Karplus & Diederichs, 2012 ▸). The merging statistics indicated that all ‘before’ and ‘after’ data sets are of high quality, with high completeness, high CC_1/2_, high 〈*I*/σ(*I*)〉 and low *R*_meas_ values (Tables 1 ▸ and 2 ▸). The variation in the numbers of sub-data sets selected for each cases (Expo. *X* or Before_*X*, After_*X*) results from the stochastic nature of the GA initialization. In the thaumatin cases, the increasing number of sub-data sets used to obtain a full data set might be due to degradation of the individual sub-data-set quality owing to nonspecific radiation damage, *i.e.* more sub-data sets are required for equivalent data quality. The lack of completeness at low resolution (inner shell) of Expo. 5 and Expo. 6 for thaumatin could be attributed to an orientation bias of the crystal because of the sample holder that was used and the fact that only small oscillations are performed. High-resolution limits were selected based on the statistics of the last data set (‘Expo. 6’ for thaumatin and ‘After_UV’ for insulin), and the same resolution limits were used for all other data sets.

For each final data set, the selection of which sub-data sets to merge was performed using a genetic algorithm. This accounts for some of the variability in the statistics between successive data sets. Furthermore, because some orientations of crystals are preferred because of the harvesting method (crystals mounted on meshes), this can lead to lower completeness in some cases. For later data sets this, in combination with the fact that completeness is weighted less heavily than 〈*I*/σ(*I*)〉 and *R*_meas_ in the GA, led to a reduction in the completeness (in all resolution shells), but with a concomitant increase in multiplicity and 〈*I*/σ(*I*)〉. This could be owing to crystals in less common orientations not being selected by the GA because of lower average 〈*I*/σ(*I*)〉 values resulting from radiation damage. Examination of sub-data sets included in Expo. 1 but missing in Expo. 5 and Expo. 6 indeed revealed lower 〈*I*/σ(*I*)〉 values and higher *R*_meas_ values.

### 3.2. RIP signal   

**Figure 1:**
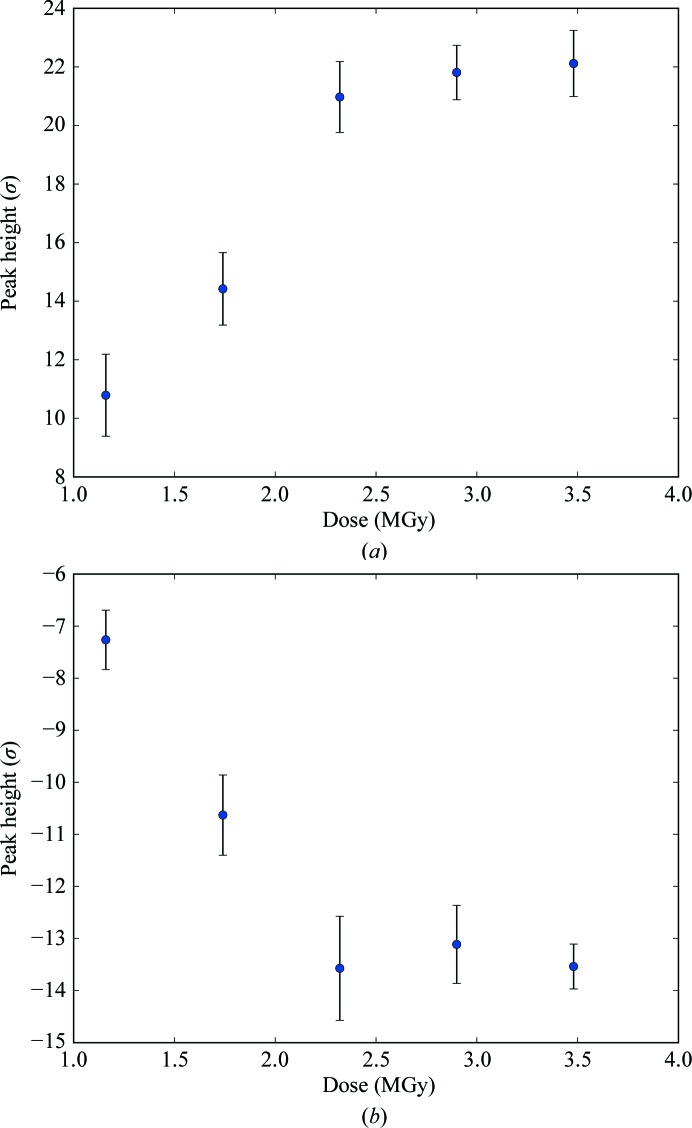
RIP peak height as a function of dose in thaumatin. (*a*) Maximum and (*b*) minimum peak heights in the model-phased *F*_before_ − *F*_after_ difference electron-density map in standard deviations above the mean. The point for each value corresponds to the average value of the peak height for all *K* values used in *SHELX*. The error bars represent the standard deviation of the peak height.

**Figure 2:**
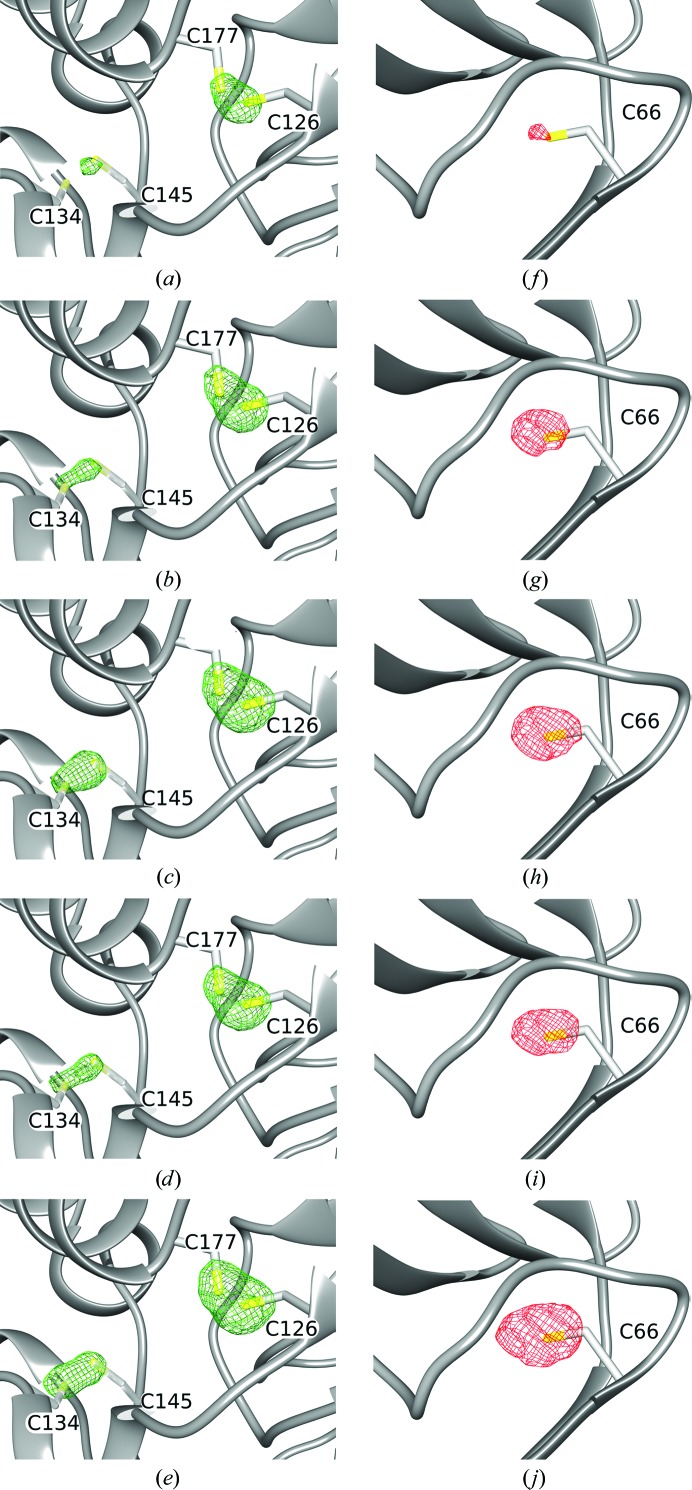
Model-phased RIP difference electron-density maps calculated for the thaumatin X-ray RIP data. (*a*)–(*e*) represent increasing dose points (Expo. 2, Expo. 3, Expo. 4, Expo. 5 and Expo. 6, respectively) subtracted from the first data set (Expo. 1). Difference density is shown as a green mesh contoured at 6σ. The disulfide bond between Cys126 and Cys177 shows the highest electron density. (*f*)–(*j*) are the same difference maps as (*a*)–(*e*) but contoured at −6.5σ in the vicinity of Cys66.

**Figure 3:**
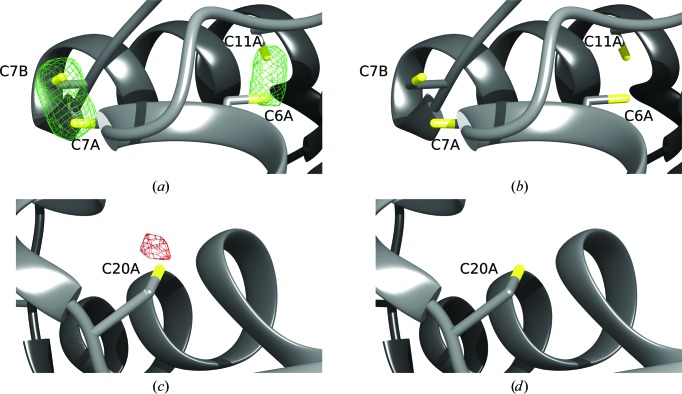
Model-phased RIP difference map for the cubic insulin UV-RIP experiment. Positive difference electron density contoured at 6σ is represented as a green mesh surrounding cysteine S atoms. Negative electron-density difference is contoured at −5σ as a red mesh in the vicinity of cysteine S atoms. (*a*) and (*c*) are RIP difference maps calculated between data sets Before_UV.1 and After_UV. (*b*) and (*d*) are RIP difference maps calculated between data sets Before_UV.1 and Before_UV.2, *i.e* before UV-light exposure. The X-­ray-only difference maps show little evidence of radiation damage, whereas the before UV illumination–post UV illumination difference map shows strong positive peaks at cysteine S^γ^ positions as well as a new peak appearing near Cys20.

The dispersive signal increases as a function of dose (Supplementary Fig. S2). This is an important metric of the RIP signal, but we have focused our analysis on RIP peak heights, which are a more sensitive indicator of the intensity of the RIP signal. It should be emphasized that this is a ‘post mortem’ analysis, which requires a high-quality phase set. In order to determine RIP peak heights, model phases are used to calculate an *F*_before_ − *F*_after_ difference map using the scaled *F*_A_ (the structure-factor amplitudes for the substructure atoms) values from *SHELXC*. This difference map is then searched for peaks. The location of the peaks reveals which atoms in the structure are damaged, and the peak height indicates the magnitude of the damage and thereby the strength of the RIP signal. In the thaumatin X-ray RIP experiment, the strongest peaks can be found over the Cys126 S atom. Fig. 1 ▸ depicts the average maximum peak heights as a function of dose. A large amount of RIP signal is present, even at relatively modest doses (for example 1.16 MGy). This signal increases dramatically when the dose is increased to 1.74 and 2.32 MGy, but only modest gains are observed above this dose (Figs. 1 ▸ *a* and 2 ▸ *a*–2 ▸ *e*). Negative peaks can also occur in a RIP difference map, which correspond to the shifting of atoms to new positions. A well known example of this is the movement of the S^γ^ position in a disulfide bond to a new position. These negative peaks are generally of a lower magnitude than the positive peaks, probably because when an S^γ^ is in a disulfide there are fewer possible rotamers than without the thiol linkage. Inspection of negative peaks in the difference map nevertheless also reveals large peaks: up to 14.24 standard deviations above the mean difference density (Figs. 2 ▸ *f*–2 ▸ *j*). Although there was no evidence of anomalous signal in the merging statistics, we calculated anomalous peak heights using *ANODE* but found that there were no peaks above 4.8 standard deviations above the mean density value. Therefore, no RIPAS (RIP with anomalous scattering) analysis was performed. For the UV RIP experiment, in order to distinguish between X-ray and UV damage, a second set of sub-data sets was collected before UV exposure (control). The average RIP peak height between the first two X-ray data sets (Before_UV.1 and Before_UV.2 in Table 2 ▸) was 4.24 standard deviations above the mean, showing that there was very little X-ray radiation damage between these data sets (Figs. 3 ▸ *b* and 3 ▸ *d*). However, comparing the third data set (After_UV in Table 2 ▸, which occurred after UV-LED exposure and had the same data-collection parameters and dose as the previous two data sets) with the first data set (Before_UV.1) revealed significant peaks in the RIP maps (Figs. 3 ▸ *a* and 3 ▸ *c*). The maximum and minimum peak heights were 23.34 and −8.99 standard deviations above the mean, respectively, with the largest differences over Cys7 and Cys20 around the Cys S^γ^ atom. As in the X-ray RIP experiment, there was very little anomalous signal, with the highest peak being 6.7 standard deviations above the mean density value.

### 3.3. Substructure determination   

Determination of RIP substructures can be difficult owing to the generally large number of atoms in the radiation-damage substructure. Indeed, one of the primary heuristics used in experimental phasing with *SHELXD*, analysis of the plot of CC(all) *versus* CC(weak), is of limited use for RIP except in very high signal cases (Supplementary Fig. S3). However, one metric of substructure-solution success that can be applied *a posteriori* is to compare experimental substructures with a pseudo-atom reference substructure. The pseudo-atom substructure was calculated with *SHELXC* and *ANODE* using the highest RIP peak heights and the refined model. Peaks above the threshold value of six standard deviations above the mean difference value are retained. This reference can then be compared with the final substructures produced by *SHELXD*. Comparison of the reference and the experimentally determined substructures results in a percentage correctness. For cubic insulin the reference contained six positive and negative sites, while for thaumatin there were 14 positive and negative sites.

**Figure 4:**
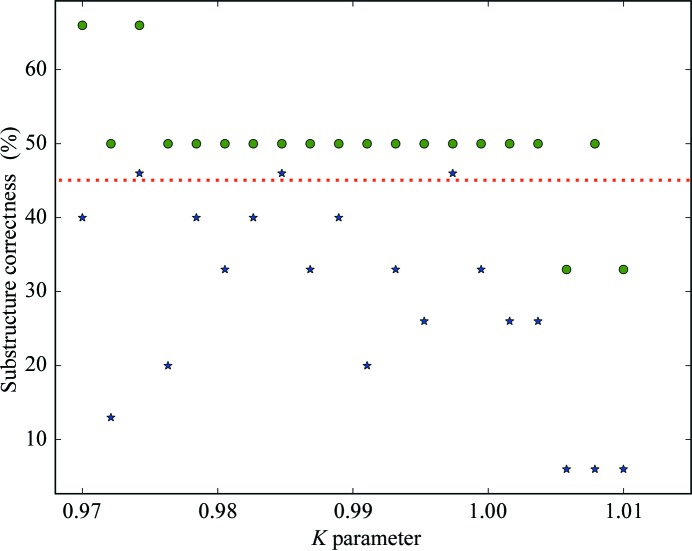
Quality of substructure determination with insulin and thaumatin data sets. The correctness of the substructure is expressed as the percentage of conserved sites in the experimental substructure compared with the reference structure (the reference model was determined by identifying peaks in a model-phased RIP difference map). Green dots correspond to the cubic insulin substructures. Blue stars correspond to thaumatin substructures for the highest dose (3.48 MGy). Below the red dashed line, the substructure correctness is less than 45%.

**Figure 5:**
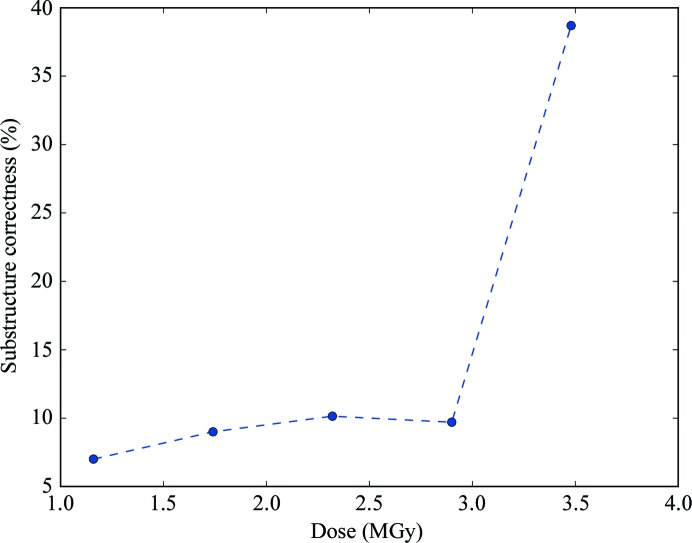
Quality of substructure determination of thaumatin as a function of X-ray dose. For each dose, the best substructure from a range of *K* values is compared against the reference. Calculation of the substructure correctness is performed as described previously. Below and including 2.9 MGy the substructure is not determinable.

Both thaumatin (X-ray RIP) and cubic insulin (UV RIP) produced substructures that could be used to produce interpretable phases. Because we have previously shown that down-weighting of the after data-set intensities after an initial scaling can improve all steps of RIP phasing, we evaluated a range of *K* values (Nanao *et al.*, 2005 ▸; de Sanctis *et al.*, 2016 ▸; Zubieta & Nanao, 2016 ▸). Because *SHELXC*/*D*/*E* were conceived for pipelines, it is feasible to evaluate a large number of *K* values automatically *via* a simple script. For each *K* value, we determined the percentage of substructure correctness as described above, as well as its average across all *K* values (average substructure correctness). For cubic insulin, the average substructure correctness was 57.67% (Fig. 4 ▸). For the most favourable thaumatin dose (3.48 MGy), the average substructure correctness was 29.47% (Figs. 4 ▸ and 5 ▸). While the quality of insulin substructures was uniformly high and was relatively unaffected by the scaling factor *K*, the thaumatin substructures could be greatly improved by applying *K* values of 0.97421, 0.98474 and 0.99737, which produced 46% correct substructures compared with 6% at *K* = 1.01 (Fig. 4 ▸). Interestingly, despite the small differences in RIP difference-map peak height at the higher doses (Fig. 1 ▸), only the highest dose data set produced correct substructures for thaumatin (Fig. 5 ▸ and Supplementary Fig. S4). For thaumatin, we used a 〈*d*′/σ(*d*′)〉 value of 1.3–1.5 to determine the high-resolution cutoff in *SHELX*. However, using one of the best *K* values (0.97421) and re-running the same *SHELXD* substructure determination at different maximal resolutions, we found that the optimal resolution cutoff appeared around 2.8–3.5 Å. This corresponds to 〈*d*′/σ(*d*′)〉 values of 2–2.5 (Supplementary Fig. S5). This reinforces the notion that rather than relying solely on a cutoff based on difference statistics, it is sometimes advisable to try different resolution cutoffs. Because of the strong RIP signal in cubic insulin, the entire positive substructure was determined across all runs from 1.5 to 4.0 Å.

### 3.4. Phase calculation   

**Figure 6:**
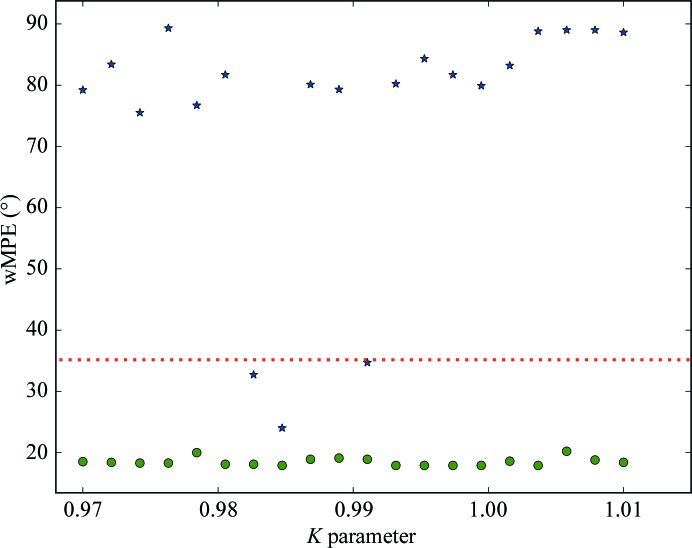
Phase errors of experimental phasing as a function of the scaling factor *K*. The wMPE is the best phase error compared with a refined model. Green dots correspond to cubic insulin and blue stars correspond to thaumatin for a dose of 3.48 MGy. The red dashed line indicates a phase error of 35°, below which maps are of excellent quality.

**Figure 7:**
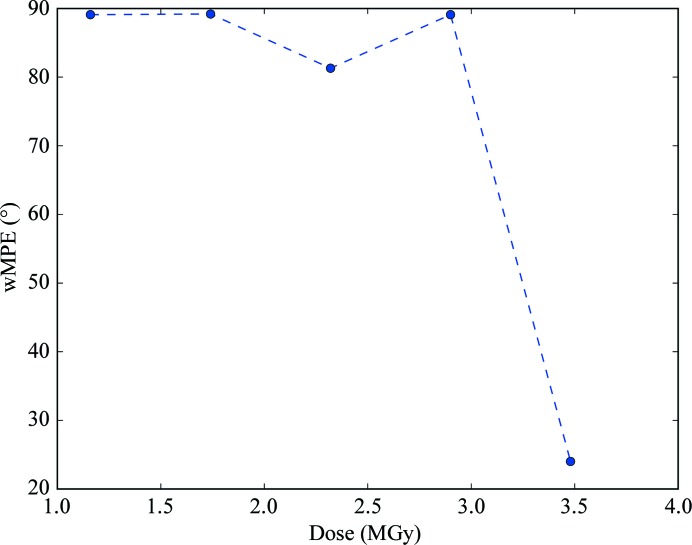
Phase errors of X-ray RIP experimental phasing of thaumatin as a function of dose.

RIP phasing proceeds in a manner similar to SIR, with the major difference being the existence of negatively occupied sites. Since no substructure-determination programs can currently determine substructures that include both positively and negatively occupied sites, the full substructure must be obtained by bootstrapping. This can be performed iteratively by rounds of phase improvement and the identification of peaks (positive and negative) in difference Fourier maps. In RIP, this process can be critical because of the starting incompleteness of the substructure (Nanao *et al.*, 2005 ▸). However, the signal in the cubic insulin UV RIP was high enough to show very little dependency on scaling *K* (Fig. 4 ▸), which has previously been observed for other UV RIP experiments (Nanao & Ravelli, 2006 ▸). Weighted mean phase errors (wMPEs) calculated from the phases determined in *SHELXE* using the final bootstrapped substructure compared with a refined model were uniformly excellent, with an average wMPE across all *K* of 18.5° (Fig. 6 ▸). As has previously been observed for other phasing methods, solution of the structure is likely when the correlation coefficient of the partially automatically built *SHELXE* model exceeds 25% and the average number of residues per fragment is greater than 10 residues. By contrast, the phase calculation for thaumatin is more sensitive to *K* values. At even the highest dose (3.48 MGy), only a few values yielded interpretable electron-density maps (Fig. 6 ▸). Phasing analysis was only performed at this dose point in view of this difficulty in phasing even with substructures that were approximately four times more complete than lower dose points (Fig. 5 ▸). Interestingly, despite the fact that the RIP peak height flattened out at a dose of 2.3 MGy, phasing and substructure determination were not successful at this dose or even at 2.9 MGy, but only at 3.48 MGy (Fig. 7 ▸ and Supplementary Fig. S6).

### 3.5. Influence of multiplicity   

Obtaining data sets with high multiplicity and completeness is at odds with controlling radiation damage. For this reason, especially in cases of small crystals and/or low symmetry, it can be difficult to obtain the two complete data sets required for X-ray RIP from a single crystal (de Sanctis & Nanao, 2012 ▸). Therefore, RIP has not been able to benefit from the advantages to phasing of high-multiplicity data sets (Usón *et al.*, 2003 ▸; Pike *et al.*, 2016 ▸). Because SSX RIP multiplicity is limited only by the diversity and number of crystals, SSX offers the possibility of obtaining much higher multiplicity data sets for both ‘damaged’ and ‘undamaged’ states. We therefore were interested in the effect of multiplicity on the various metrics and stages of phasing. For these analyses we started with the very high multiplicity data sets discussed earlier (thaumatin Expo. 1 and 6, and insulin Before_UV.1 and After_UV) and reduced their multiplicity incrementally to create new data sets (Tables 3 ▸ and 4 ▸). While there are many potential strategies to reduce the multiplicity, such as decreasing the number of images in each sub-data set or removing sub-data sets based on specific criteria such as *I*/σ(*I*), we have taken a practical approach to the reduction of multiplicity and randomly omitted sub-data sets. Enough data sets were removed incrementally to reduce the multiplicity by 1.5–2-fold at a time. Furthermore, in order to reduce the effects of resolution, we used the same resolution range for all data sets, even if it produced poor statistics in some of the outer resolution shells.

#### 3.5.1. The effect of multiplicity on RIP signal and phasing   

**Figure 8:**
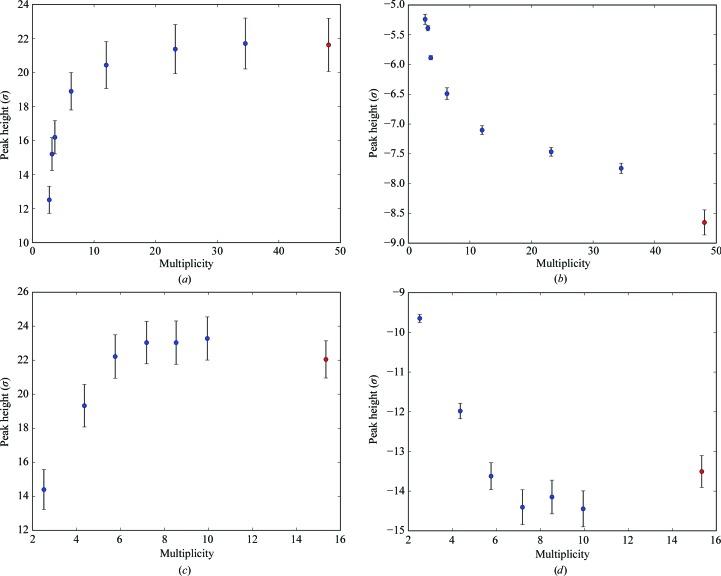
The effect of artificially reducing data-set multiplicity on average model-phased RIP difference-map peak height. (*a*) and (*b*) correspond to the maximum and minimum peak heights in the model-phased *F*_before_ − *F*_after_ difference electron-density map for the cubic insulin UV RIP data. (*c*) and (*d*) correspond to the maximum and minimum peak heights for the thaumatin X-ray RIP data. Peak heights are averaged across all *K* values. Red points correspond to the original data set without multiplicity reduction. The error bars represent the standard deviation of the peak heights across different *K* values for scaling.

**Figure 9:**
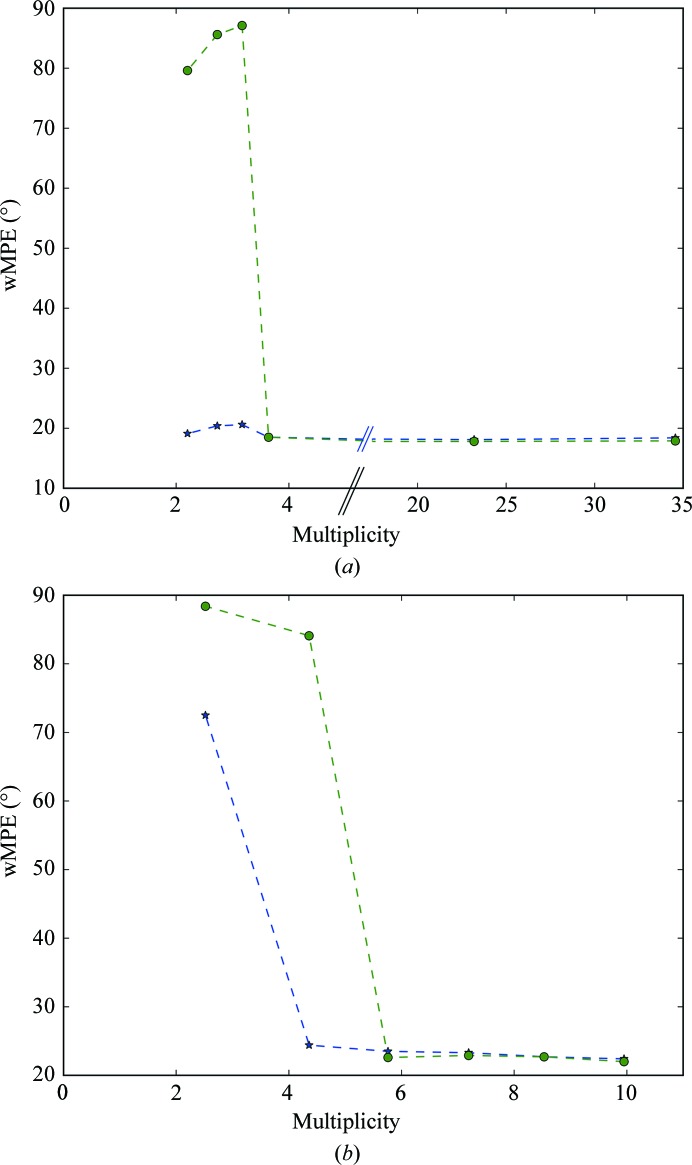
Experimental phasing for insulin UV RIP (*a*) and thaumatin X-ray RIP (*b*) starting from the known (blue stars) or experimentally determined substructures (green circles). The best wMPE across all trials is reported compared with a refined model.

In cubic insulin, the effect of multiplicity is readily apparent. In previous RIP experiments, multiplicities of approximately fourfold to 1.5 Å resolution (Nanao *et al.*, 2005 ▸) were typically achieved. Because multiple crystals can be used in SSX, the multiplicity can be greatly increased. In particular, we observed exponential gains in RIP peak signal, as assessed by the maximal peak height in RIP difference maps, up to 12-fold multiplicity for insulin (Figs. 8 ▸ *a* and 8 ▸ *b*) and up to sevenfold multiplicity for thaumatin (Figs. 8 ▸ *c* and 8 ▸ *d*). These gains can be seen in both positive and negative peak heights. The point of diminishing returns occurs around 25-fold multiplicity for insulin and eightfold multiplicity for thaumatin. This trend continues into phase determination. A threshold of signal strength occurs at a multiplicity of four for cubic insulin (UV RIP; Figs. 9 ▸ *a* and 9 ▸ *b*). As has been seen for other phasing methods, there is a ‘grey area’ where there is sufficient signal to determine interpretable phases, but there is not sufficient signal to determine correct RIP substructures. In other words, if the known substructure is used as a starting point in *SHELXE* then phasing succeeds, but obviously this is an artificial situation. For thaumatin X-ray RIP, we observed a similar shift in the multiplicity requirements for substructure determination and phasing: substructure determination and phasing required a multiplicity of six, which could be reduced to four when starting from the known substructure.

## 4. Discussion   

RIP offers a complementary method to traditional anomalous and isomorphous methods for the experimental determination of phases. Although RIP can also be used in combination with anomalous and isomorphous methods, it is a useful method on its own, particularly when heavy-atom derivatization or seleno­methionine substitution is difficult. Recent advances in multiple-crystal techniques have made it practical to determine high-resolution structures from X-ray data acquired from a large number of crystals. Here, we show that a serial approach yields sufficient signal to determine phases *de novo* by both X-ray RIP and UV RIP for these two test systems. In this study, we have assembled low-dose and high-dose data sets independently; however, we are also exploring the possibility of improving RIP signal by optimizing both data sets simultaneously. In this way, the isomorphous signal could be improved depending on which sub-data sets are selected. Because of the relatively high symmetry of thaumatin and insulin, we have simply used the strongest sub-data set as a reference for indexing other sub-data sets during processing. However, in some cases an individual sub-data set might not contain enough reflections for this purpose. In these cases, alternate methods for indexing (and resolving indexing ambiguity) might become necessary, for example using the method developed by Brehm & Diederichs (2014 ▸). For very incomplete sub-data sets, scaling becomes impossible owing to a lack of common reflections, unless a reference data set is available.

For X-ray RIP, we show that improvements can be made to the RIP signal up to 4 MGy. This suggests a guideline for the design of serial RIP experiments. For example, at the ESRF this information can be easily used in the *MeshAndCollect* workflow within *MXCuBE* (Gabadinho *et al.*, 2010 ▸; Zander *et al.*, 2015 ▸). Specifically, once the diffractive map has been constructed, an estimation of dose rate is provided and the user can modify not only the individual data-collection parameters but also the number of times that each position is re-collected. The user could therefore change the experimental parameters to provide 1 MGy per sub-data set, and collect each position four times. Aside from the ease of the experiment, one key advantage of the serial approach is that much higher data quality at a given dose can be achieved per final data set compared with single crystals. This increase in the number of diffraction patterns facilitates the collection of high-multiplicity data sets. High multiplicity has in turn been shown to be critical for phasing success in many types of experimental phasing, particularly SAD (Cianci *et al.*, 2008 ▸). However, because the traditional approach to RIP calls for extremely low dose ‘before’ and ‘after’ data sets, RIP has not typically benefitted from high-multiplicity data collections. Indeed, in some cases it can be challenging even to collect two complete low-multiplicity data sets. Here, we show that the serial approach can be used to produce high-multiplicity data sets with excellent statistics and furthermore that exponential increases in RIP peak heights occur as a function of multiplicity up to a point of diminishing returns of eightfold and 25-fold multiplicity for thaumatin and insulin, respectively. As has been previously shown for single-crystal X-ray RIP, initial scaling by conventional methods followed by downscaling of the high-dose data sets can significantly improve substructure solution (Nanao *et al.*, 2005 ▸). There still is no way to *a priori* find the best scale factor *K*, besides trying multiple *K* values, but the scriptability and direct support of this parameter in *SHELXC* makes the process straightforward. It is possible that other methods, such as adjusting the *K* value to maximize non-origin Patterson peak heights, might also be effective. Furthermore, while running *SHELXE* for each *K* value adds computing time, it is compensated by the calculation of two critical statistics from *SHELXE*: the correlation coefficient of the partially automatically built model against the native data and the average fragment size. These two parameters are highly predictive of phasing success for RIP, as with other phasing methods, and are the primary means by which one can evaluate the success of RIP in new systems.

Because these are test systems, we do not yet know whether these patterns will be borne out in cases of low symmetry or large non-isomorphism. It is worth noting that we have focused on the most well known X-ray-sensitive groups: disulfides. However, in future work we hope to extend this to other radiation-sensitive atoms such as oxygen atoms in carboxylates and heavy atoms such as selenium, in which the anomalous signal can be combined with the RIP signal as previously described for single crystals (Schiltz *et al.*, 2004 ▸; Ravelli *et al.*, 2005 ▸).

## Supplementary Material

Supplementary Figures.. DOI: 10.1107/S2059798318001535/di5013sup1.pdf

## Figures and Tables

**Table 1:** Thaumatin X-ray RIP sub-data-set data-collection statistics Exposures (Expo.) 1–6 were obtained by successive data collection executed through a single diffractive map determined by the *MeshAndCollect* workflow.

Data-set name	Expo. 1	Expo. 2	Expo. 3	Expo. 4	Expo. 5	Expo. 6
Space group	*P*4_1_2_1_2	*P*4_1_2_1_2	*P*4_1_2_1_2	*P*4_1_2_1_2	*P*4_1_2_1_2	*P*4_1_2_1_2
*a*, *b*, *c* (Å)	58.31, 58.31, 150.98	58.33, 58.33, 151.13	58.42, 58.42, 151.06	58.43, 58.43, 151.21	58.52, 58.52, 151.34	58.31, 58.31, 150.96
α, β, γ (°)	90, 90, 90	90, 90, 90	90, 90, 90	90, 90, 90	90, 90, 90	90, 90, 90
Cumulative dose per sub-data set (MGy)	0.72	1.16	1.74	2.32	2.90	3.48
No. of sub-data sets (100 crystals collected)	22	25	24	36	33	32
Resolution range (Å)						
Overall	100–1.40	100–1.40	100–1.40	100–1.40	100–1.40	100–1.40
Inner shell	100–6.26	100–6.26	100–6.26	100–6.26	100–6.26	100–6.26
Outer shell	1.44–1.40	1.44–1.40	1.44–1.40	1.44–1.40	1.44–1.40	1.44–1.40
Total No. of reflections						
Inner shell	9601	10575	10527	15744	14777	13935
Overall	806228	915459	878916	1324552	1219289	1172721
Outer shell	57767	65557	62992	94768	87347	83749
No. of unique reflections						
Overall	52243	50116	50825	52478	48493	48745
Inner shell	706	653	677	707	548	632
Outer shell	3788	3656	3670	3798	3708	3535
Completeness (%)						
Inner shell	99.9	92.4	95.6	99.6	76.9	89.4
Outer shell	100.0	96.2	96.3	99.7	96.8	93.4
Overall	99.9	95.7	96.8	99.8	91.9	93.3
Multiplicity						
Inner shell	13.59	16.19	15.55	22.27	26.96	22.04
Outer shell	15.25	17.93	17.16	24.95	23.55	23.69
Overall	15.34	18.26	17.29	25.24	25.14	24.06
*R*_merge_ † (%)						
Inner shell	5.6	5.4	6.1	5.5	5.6	5.2
Outer shell	224.0	218.2	222.6	262.7	262.4	276.1
Overall	18.1	21.2	17.6	20.3	18.7	20.5
*R*_meas_ † (%)						
Inner shell	5.8	5.5	5.6	5.6	5.8	5.3
Outer shell	231.8	224.4	229.2	268.0	268.0	281.8
Overall	18.7	21.8	18.1	20.7	19.1	20.9
〈*I*/σ(*I*)〉						
Inner shell	36.61	39.29	41.91	45.28	55.72	48.90
Outer shell	1.06	1.22	1.22	1.10	1.16	1.10
Overall	10.59	11.87	12.15	12.71	13.58	13.38
CC_1/2_ ‡ (%)						
Inner shell	99.9*	99.9*	99.9*	99.9*	99.9*	99.9*
Outer shell	28.1*	35.2*	38.7*	28.2*	31.6*	33.1*
Overall	99.8*	99.7*	99.9*	99.8*	99.9*	99.9*
Anomalous correlation coefficient						
Inner shell	9	14	3	13	5	14
Outer shell	0	−2	0	−1	3	−1
Overall	−1	−2	0	0	1	1
SigAno						
Inner shell	0.800	0.893	0.844	0.945	0.896	0.978
Outer shell	0.660	0.663	0.682	0.664	0.688	0.651
Overall	0.769	0.773	0.784	0.780	0.790	0.785

† *R*_merge_ = 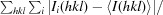 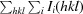 and *R*_meas_ = 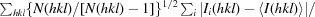 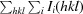.

‡ CC_1/2_ values that are significant at the 0.1% level are marked by an asterisk.

**Table 2:** Cubic insulin UV RIP sub-data-set data-collection statistics Before_UV.1 is the first data set obtained before UV-light exposure. Before_UV.2 is a second data set, without UV light to control for the effects of X-ray damage. After_UV is the data set obtained after UV-light exposure. Note that not all data sets from the *MeshAndCollect* procedure were used. For each final data set, the selection of sub-data sets to merge was performed using a genetic algorithm.

Data-set name	Before_UV.1	Before_UV. 2	After_UV
Space group	*I*2_1_3	*I*2_1_3	*I*2_1_3
*a*, *b*, *c* (Å)	78.92, 78.92, 78.92	78.78, 78.78, 78.78	78.88, 78.88, 78.88
α, β, γ (°)	90, 90, 90	90, 90, 90	90, 90, 90
Cumulative dose per sub-data set (MGy)	0.43	0.86	1.29
No. of sub-data sets	91	76	88
Resolution range (Å)			
Overall	100–1.4	100–1.4	100–1.5
Inner shell	100–6.26	100–6.26	100–6.71
Outer shell	1.44–1.40	1.44–1.40	1.54–1.50
Total No. of reflections			
Inner shell	18850	15562	14660
Overall	1616572	1333165	1219845
Outer shell	121080	98710	91770
No. of unique reflections			
Inner shell	355	356	295
Overall	31315	31275	25409
Outer shell	2358	2336	1926
Completeness (%)			
Inner shell	99.7	100.0	100.0
Outer shell	100.0	100.0	100.0
Overall	100.0	100.0	100.0
Multiplicity			
Inner shell	53.09	43.71	49.69
Outer shell	51.34	42.25	47.65
Overall	51.62	42.62	48.08
*R*_merge_ (%)			
Inner shell	12.7	13.5	18.0
Outer shell	318.4	394.6	522.6
Overall	22.5	25.1	50.0
*R*_meas_ (%)			
Inner shell	12.8	13.7	18.2
Outer shell	321.6	399.4	528.1
Overall	22.7	25.4	50.5
〈*I*/σ(*I*)〉			
Inner shell	52.63	47.62	36.45
Outer shell	2.21	1.64	1.62
Overall	18.67	16.47	13.45
CC_1/2_ † (%)			
Inner shell	100.0*	99.7*	99.8*
Outer shell	71.0*	58.6*	57.1*
Overall	99.9*	99.8*	99.8*
Anomalous correlation coefficient			
Inner shell	29	24	10
Outer shell	−7	1	−2
Overall	1	2	−1
SigAno			
Inner shell	1.199	1.082	0.943
Outer shell	0.706	0.723	0.696
Overall	0.821	0.817	0.783

† CC_1/2_ values that are significant at the 0.1% level are marked by an asterisk.

**Table 3:** Thaumatin X-ray RIP overall data-collection statistics after multiplicity reduction Each data set has had its multiplicity artificially reduced compared with the original data set (Expo. 1–Expo. 6 in Table 1 ▸) by removing enough images to reduce the multiplicity by 1.5–2-fold. For the After series, a larger number of sub-data sets are used compared with the Before series, because the starting full data sets also required more sub-data sets to achieve 〈*I*/σ(*I*)〉 values comparable to the earlier dose points, possibly because of a degradation in data quality after X-ray damage. The same resolution ranges were used for all data sets, which caused the outer shell statistics to degrade in some cases.

	X-ray RIP											
Data-set name	Before_*A*	Before_*B*	Before_*C*	Before_*D*	Before_*E*	Before_*F*	After_*A*	After_*B*	After_*C*	After_*D*	After_*E*	After_*F*
No. of sub-data sets	14	12	10	8	6	3	24	22	20	18	16	13
Resolution range (Å)												
Overall	100–1.4	100–1.4	100–1.4	100–1.4	100–1.4	100–1.4	100–1.4	100–1.4	100–1.4	100–1.4	100–1.4	100–1.4
Outer shell	1.44–1.40	1.44–1.40	1.44–1.40	1.44–1.40	1.44–1.40	1.44–1.40	1.44–1.40	1.44–1.40	1.44–1.40	1.44–1.40	1.44–1.40	1.44–1.40
Total No. of reflections												
Overall	513560	440049	366446	292681	219403	109888	879971	806757	733172	660329	586699	477028
Outer shell	36745	31479	26224	20990	15743	7911	62810	57587	52356	47156	41926	34088
No. of unique reflections												
Overall	51569	51534	50962	50817	50307	43461	48742	48736	48597	48596	47950	47937
Outer shell	3704	3704	3698	3679	3631	3083	3535	3534	3518	3518	3484	3484
Completeness (%)												
Inner shell	97.6	97.6	96.3	96.0	96.0	80.8	89.4	89.4	89.3	89.3	85.9	85.9
Outer shell	97.8	97.8	97.6	97.1	95.9	81.4	93.4	93.4	93.0	93.0	92.1	92.1
Overall	98.6	98.6	97.5	97.2	96.2	83.1	93.2	93.2	93.0	93.0	91.7	91.7
Multiplicity												
Inner shell	8.90	7.71	6.42	5.12	3.82	2.24	16.47	15.16	13.84	12.43	11.50	9.32
Outer shell	9.90	8.49	7.09	5.70	4.33	2.56	17.76	16.29	14.88	13.40	12.03	9.78
Overall	9.95	8.53	7.19	5.76	4.36	2.52	18.05	16.55	15.08	13.58	12.23	9.95
*R*_merge_ (%)												
Inner shell	5.5	5.5	5.7	5.4	5.6	4.6	5.1	5.1	5.0	4.9	5.0	4.8
Outer shell	201.3	199.1	244.9	184.0	205.7	174.7	278.9	273.9	285.9	279.9	284.5	263.6
Overall	17.2	17.1	17.7	16.3	17.0	14.7	20.4	20.3	20.8	20.8	20.9	19.5
*R*_meas_ (%)												
Inner shell	5.8	5.9	6.1	6.0	6.5	5.7	5.2	5.2	5.2	5.2	5.2	5.1
Outer shell	212.2	211.9	219.8	202.1	232.4	211.0	286.5	282.3	295.3	290.2	296.2	277.1
Overall	18.1	18.2	19.0	17.9	19.2	17.7	21.0	20.9	21.5	21.5	21.8	20.4
〈*I*/σ(*I*)〉												
Inner shell	31.49	29.18	26.70	23.90	18.85	15.14	45.53	41.84	40.02	38.66	37.64	35.12
Outer shell	0.98	0.93	0.85	0.82	0.61	0.48	0.94	0.92	0.85	0.82	0.77	0.75
Overall	9.13	8.51	7.74	7.05	5.53	4.25	11.72	11.32	10.69	10.27	9.77	9.20
CC_1/2_ † (%)												
Inner shell	99.8*	99.8*	99.8*	99.8*	99.7*	99.4*	99.9*	99.9*	99.9*	99.9*	99.9*	99.8*
Outer shell	23.4*	22.7*	18.8*	18.3*	12.7*	10.9*	28.3*	29.2*	28.3*	26.3*	23.3*	22.5*
Overall	99.7*	99.7*	99.6*	99.5*	99.3*	99.2*	99.8*	99.8*	99.8*	99.8*	99.8*	99.8*

† CC_1/2_ values that are significant at the 0.1% level are marked by an asterisk.

**Table:** Each original data set (Before_UV.1–After_UV; Table 2 ▸) has its maximal multiplicity artificially reduced compared with the starting data set. For the After series, a larger number of sub-data sets are used compared with the Before series, because the starting full data sets also required more sub-data sets to achieve 〈*I*/σ(*I*)〉 values comparable to the earlier dose points, possibly because of a degradation in data quality after X-ray damage. The same resolution ranges were used for all data sets, which caused the outer shell statistics to degrade in some cases.

	UV RIP							
Data-set name	Before_*Ai*	Before_*Bi*	Before_*Ci*	Before_*Di*	Before_*Ei*	Before_*Fi*	Before_*Gi*	Before_*Hi*
No. of sub-data sets	61	41	21	11	6	5	4	3
Resolution range (Å)								
Overall	100–1.4	100–1.4	100–1.4	100–1.4	100–1.4	100–1.4	100–1.4	100–1.4
Outer shell	1.44–1.40	1.44–1.40	1.44–1.40	1.44–1.40	1.44–1.40	1.44–1.40	1.44–1.40	1.44–1.40
Total No. of reflections								
Overall	1082251	726413	374875	196084	106764	89232	71445	53423
Outer shell	80952	54294	28131	31231	8075	6733	5394	4030
No. of unique reflections								
Overall	31317	31317	31291	14766	29308	28118	26159	24211
Outer shell	2358	2358	2358	2357	2279	2131	1979	1857
Completeness (%)								
Inner shell	100.0	100.0	99.2	98.9	76.1	71.1	65.4	64.9
Outer shell	100.0	100.0	100.0	100.0	97.1	90.8	84.4	79.2
Overall	100.0	100.0	100.0	99.8	93.6	89.8	83.6	77.4
Multiplicity								
Inner shell	35.72	24.05	12.44	6.53	4.59	4.13	3.58	2.63
Outer shell	34.33	23.02	11.93	6.26	3.54	3.16	2.72	2.17
Overall	34.56	23.19	11.98	6.28	3.64	3.17	2.73	2.20
*R*_merge_ (%)								
Inner shell	13.8	12.1	9.4	8.4	7.6	7.9	7.8	6.9
Outer shell	291.9	232.5	170.1	154.2	147.6	139.5	148.1	134.3
Overall	22.7	19.2	14.1	12.1	10.4	10.2	10.2	9.8
*R*_meas_ (%)								
Inner shell	14.0	12.5	9.8	9.2	8.5	9.0	9.1	8.3
Outer shell	296.3	237.7	177.9	168.6	172.3	164.0	177.9	167.0
Overall	23.0	15.19	14.8	13.2	12.0	11.9	12.1	12.0
〈*I*/σ(*I*)〉								
Inner shell	44.17	39.86	29.99	22.59	19.35	17.39	15.93	13.66
Outer shell	2.11	2.11	1.78	1.31	0.89	0.90	0.76	0.74
Overall	16.32	15.19	11.71	8.79	6.39	5.97	5.30	4.81
CC_1/2_ † (%)								
Inner shell	99.8*	99.6*	99.5*	99.2*	99.3*	98.9*	98.8*	99.5*
Outer shell	69.0*	67.0*	55.5	38.7	26.4	28.8*	27.1	25.2*
Overall	99.9*	99.8*	99.5*	99.2*	99.2*	99.1*	99.0*	99.1*

**Table:** 

	UV RIP							
Data-set name	After_*Ai*	After_*Bi*	After_*Ci*	After_*Di*	After_*Ei*	After_*Fi*	After_*Gi*	After_*Hi*
No. of sub-data sets	61	41	21	11	6	5	4	3
Resolution range (Å)								
Overall	100–1.5	100–1.5	100–1.5	100–1.5	100–1.5	100–1.5	100–1.5	100–1.5
Outer shell	1.54–1.50	1.54–1.50	1.54–1.50	1.54–1.50	1.54–1.50	1.54–1.50	1.54–1.50	1.54–1.50
Total No. of reflections								
Overall	847024	572864	299805	156750	85897	71422	56916	42477
Outer shell	63380	432243	23078	12058	6604	5477	4335	3224
No. of unique reflections								
Overall	25406	25411	25405	25388	24902	24201	23105	21081
Outer shell	1925	1926	1926	1926	1897	1848	1762	1613
Completeness (%)								
Inner shell	100.0	100.0	99.7	99.3	96.3	90.2	85.8	78.3
Outer shell	100.0	100.0	100.0	100.0	98.8	96.2	91.8	84.0
Overall	100.0	100.0	100.0	99.9	98.0	95.2	90.9	83.0
Multiplicity								
Inner shell	34.65	33.40	11.93	6.30	3.52	3.09	2.57	2.07
Outer shell	32.92	22.45	11.98	6.26	3.48	2.96	2.46	2.00
Overall	33.34	22.54	11.80	6.17	3.45	2.95	2.46	2.01
*R*_merge_ (%)								
Inner shell	18.1	14.3	13.2	13.8	8.5	8.2	8.0	7.6
Outer shell	534.3	438.7	301.8	315.2	371.6	476.4	775.5	2747.3
Overall	50.4	40.6	26.2	26.1	22.1	23.4	25.5	28.3
*R*_meas_ (%)								
Inner shell	18.5	14.7	14.1	15.1	10.0	9.8	9.8	9.7
Outer shell	542.6	448.8	315.3	344.0	436.1	572.2	959.7	3514.1
Overall	51.2	41.6	27.4	28.6	25.8	28.0	31.4	35.9
〈*I*/σ(*I*)〉								
Inner shell	31.52	29.23	22.56	17.17	13.18	11.86	10.52	9.39
Outer shell	1.49	1.48	1.32	0.95	0.62	0.52	0.32	0.12
Overall	11.89	11.05	8.91	6.60	4.78	4.16	3.43	2.47
CC_1/2_ † (%)								
Inner shell	99.8*	99.9*	78.4*	99.3*	99.2*	98.5*	98.5*	98.8*
Outer shell	53.5*	51.8*	43.5*	22.0	8.7	8.9	2.2	2.4
Overall	99.7*	99.7*	94.4*	96.1*	98.4*	97.8*	97.2*	97.1*

† CC_1/2_ values that are significant at the 0.1% level are marked by an asterisk.
