# Supplementary material for: X-ray and UV radiation-damage-induced phasing using synchrotron serial crystallography
Source: Acta Crystallogr D Struct Biol. 2018 Apr 6;74(Pt 4):366–78. doi: 10.1107/S2059798318001535 (PMC5892880; doi:10.1107/S2059798318001535)
Supplement: Supplementary file 1 [file d-74-00366-sup1.pdf]

STRUCTURAL
BIOLOGY

Volume 74 (2018)

Supporting information for article:

X-ray and UV radiation-damage phasing using synchrotron serial crystallography

Nicolas Foos, Carolin Seuring, Robin Schubert, Anja Burkhardt, Olof Svensson, Alke Meents, Henry N. Chapman and Max H. Nanao

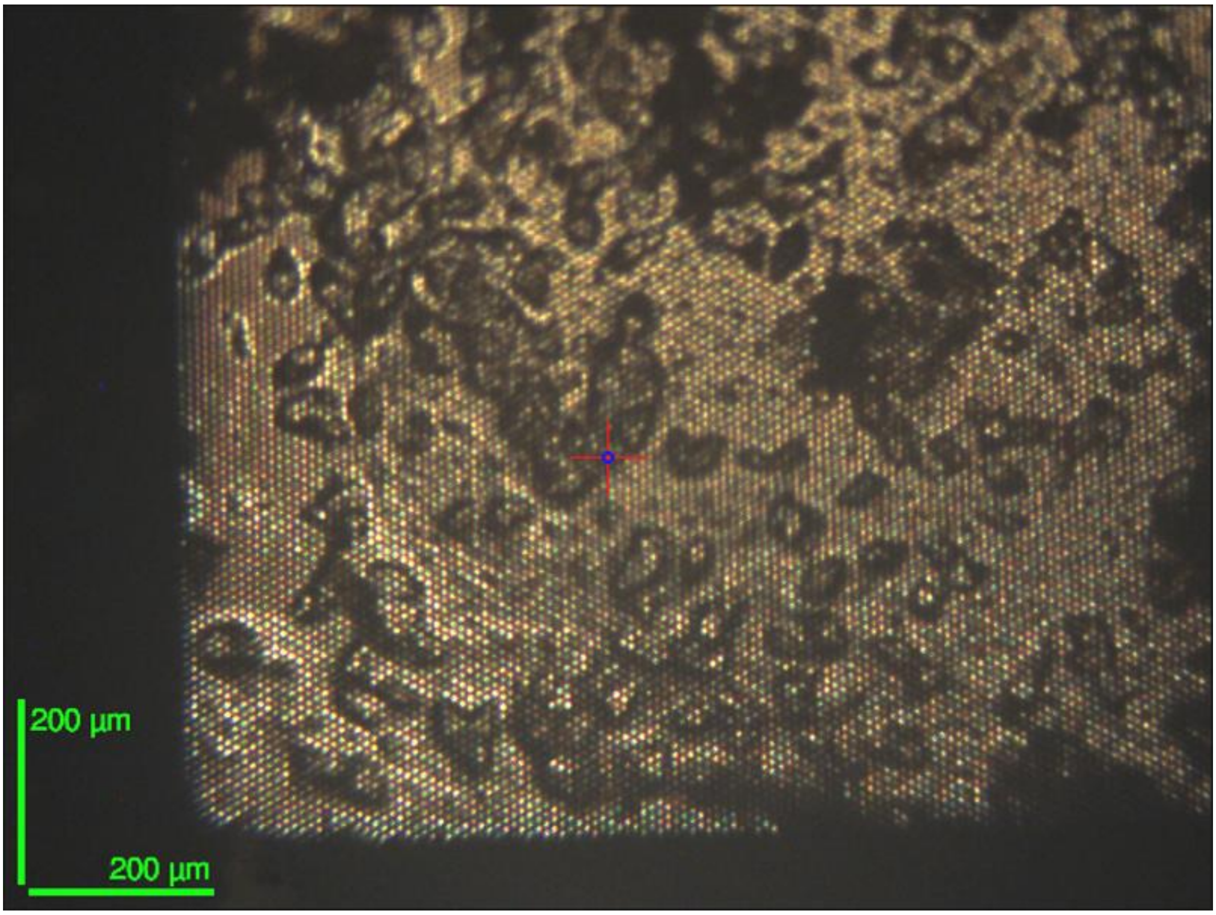

Figure S1 Snapshot of insulin microcrystals in a Si support.

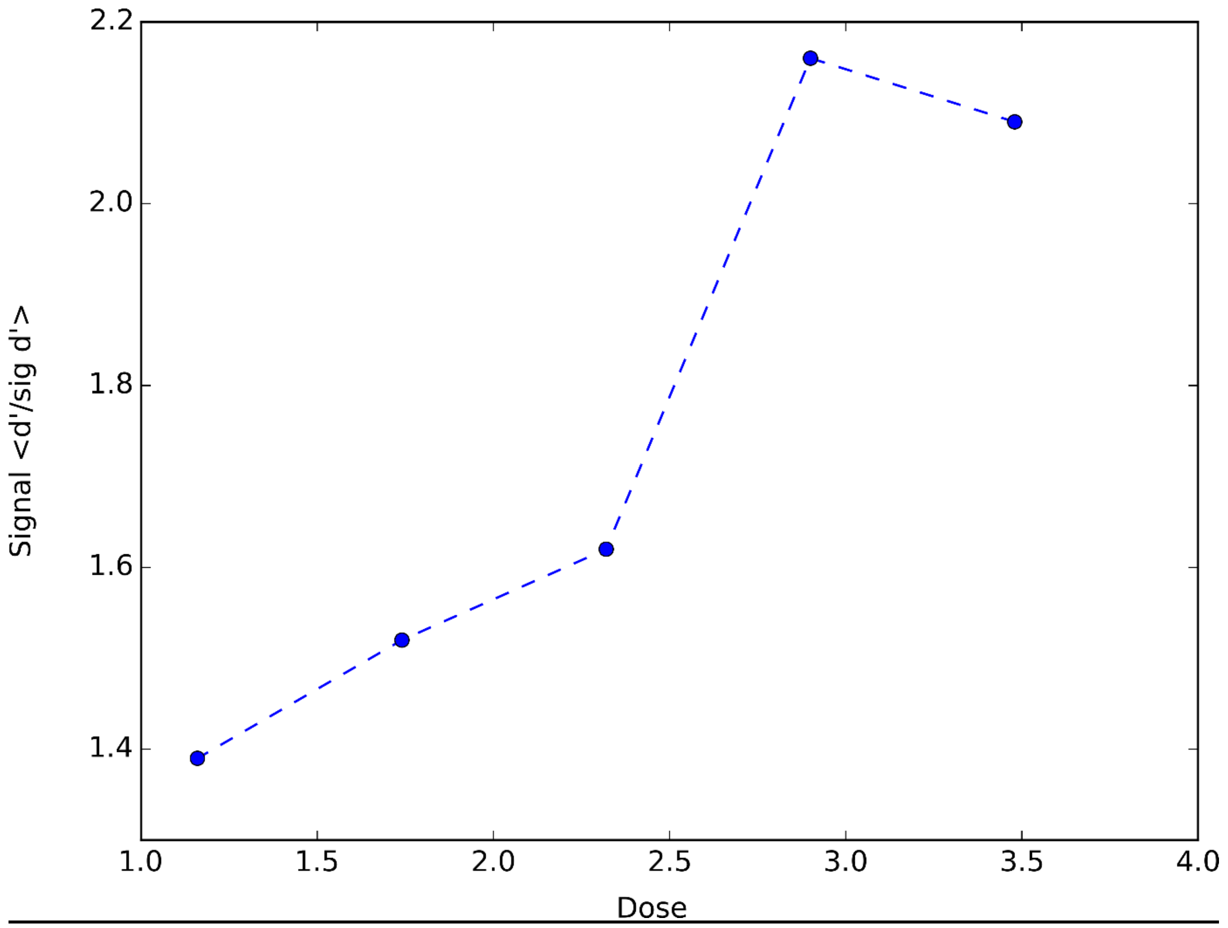

Figure S2 <d'/**σ**(d')> as a function of dose in the thaumatin X-ray RIP experiment.

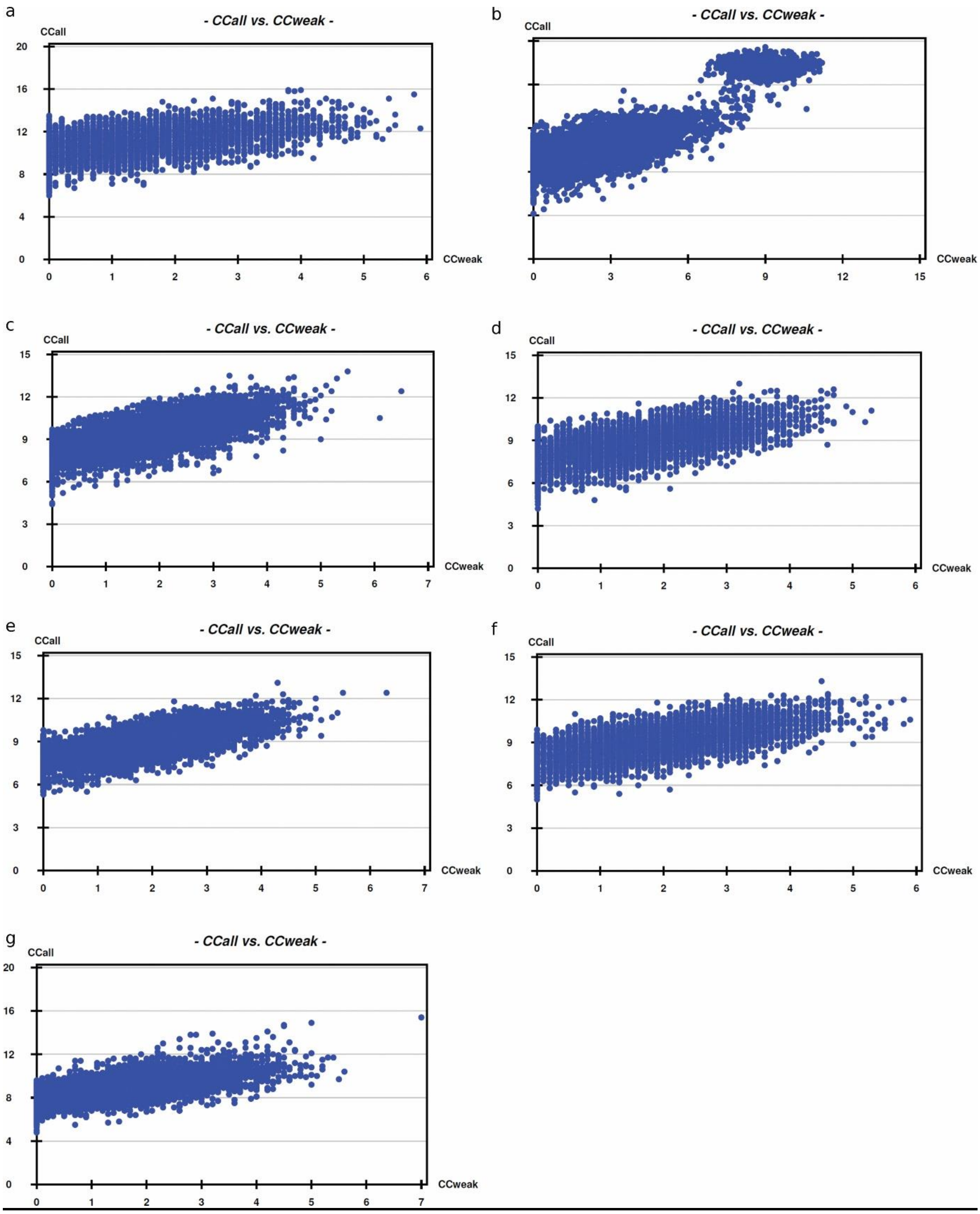

Figure S3 SHELXD scatter plots of *CCall vs. CCweak* trials. For all cases, data are shown for the K value which produced the best substructure. Panels a) and b) are insulin UV-RIP. Panels c)-g) are thaumatin X-ray RIP. a) control experiment using Before UV.1 (*derivative*) and Before UV.2 (*native*) datasets with K = 0.99105. (b) Before UV.1 (*derivative*) and After UV.1 (*native*) with K = 0.99105. For all subsequent panels, the dataset Expo. 1 is used as *derivative* and the datasets used as *native* is varied, as described in the text. c) *native* is Expo. 2 with K = 0.97842. d) *native* is Expo. 3 with K =

0.97421. e) *native* is Expo. 4 with K = 0.97632. f) native is Expo. 5 with K = 0.97632 and g) *native* is Expo. 6 with K = 0.97842.

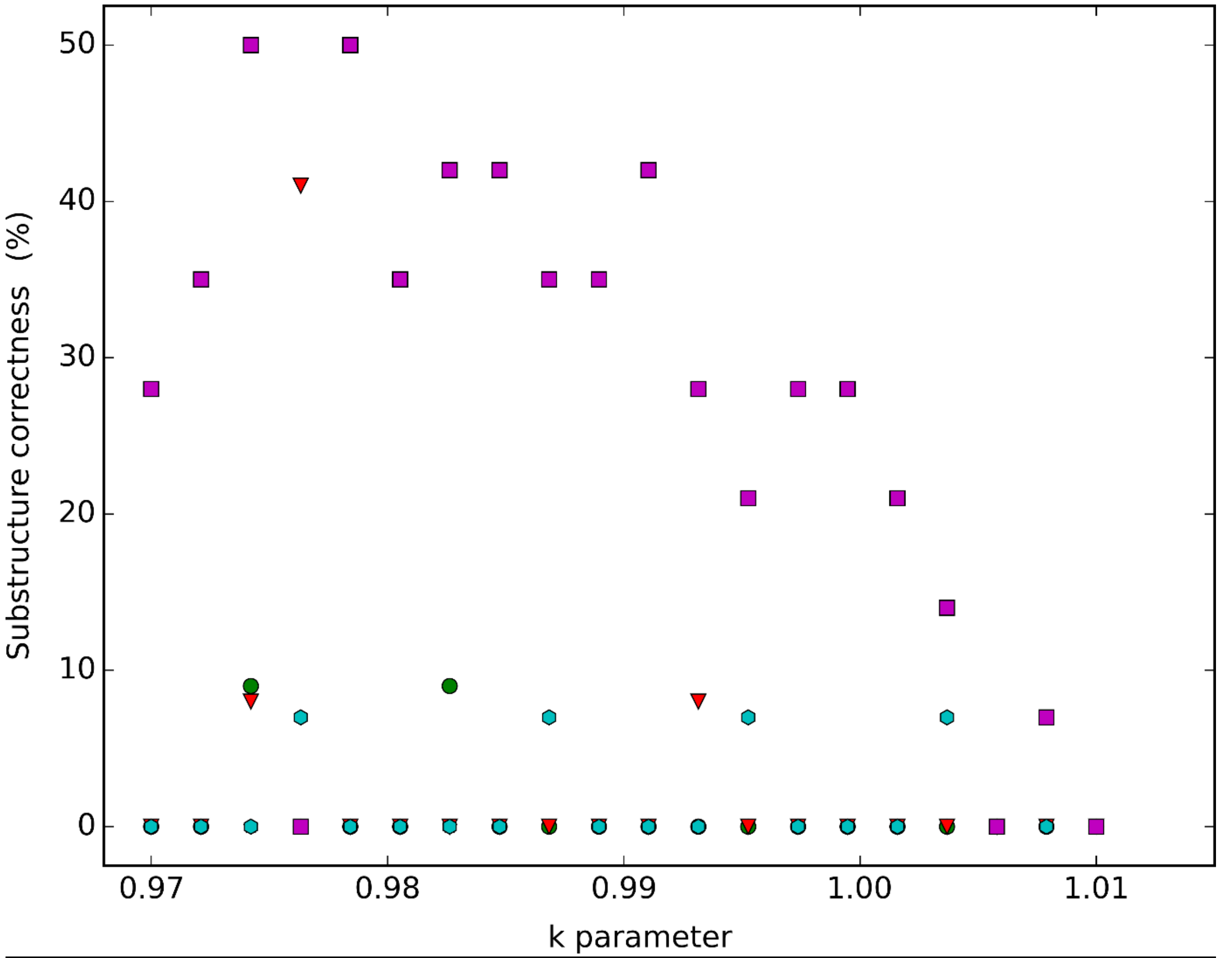

Figure S4 Quality of substructure determination of thaumatin for different doses as a function of the K scaling factor. Blue stars represent 1.16 MGy, green dots 1.74 MGy, red triangles 2.32 MGy, cyan hexagons 2.90 MGy and purple squares 3.46 MGy. Correctness of substructure is calculated as described earlier in Figure 4. Below 2.9 MGy the substructure is not determinable and the best substructures are obtained with the highest dose.

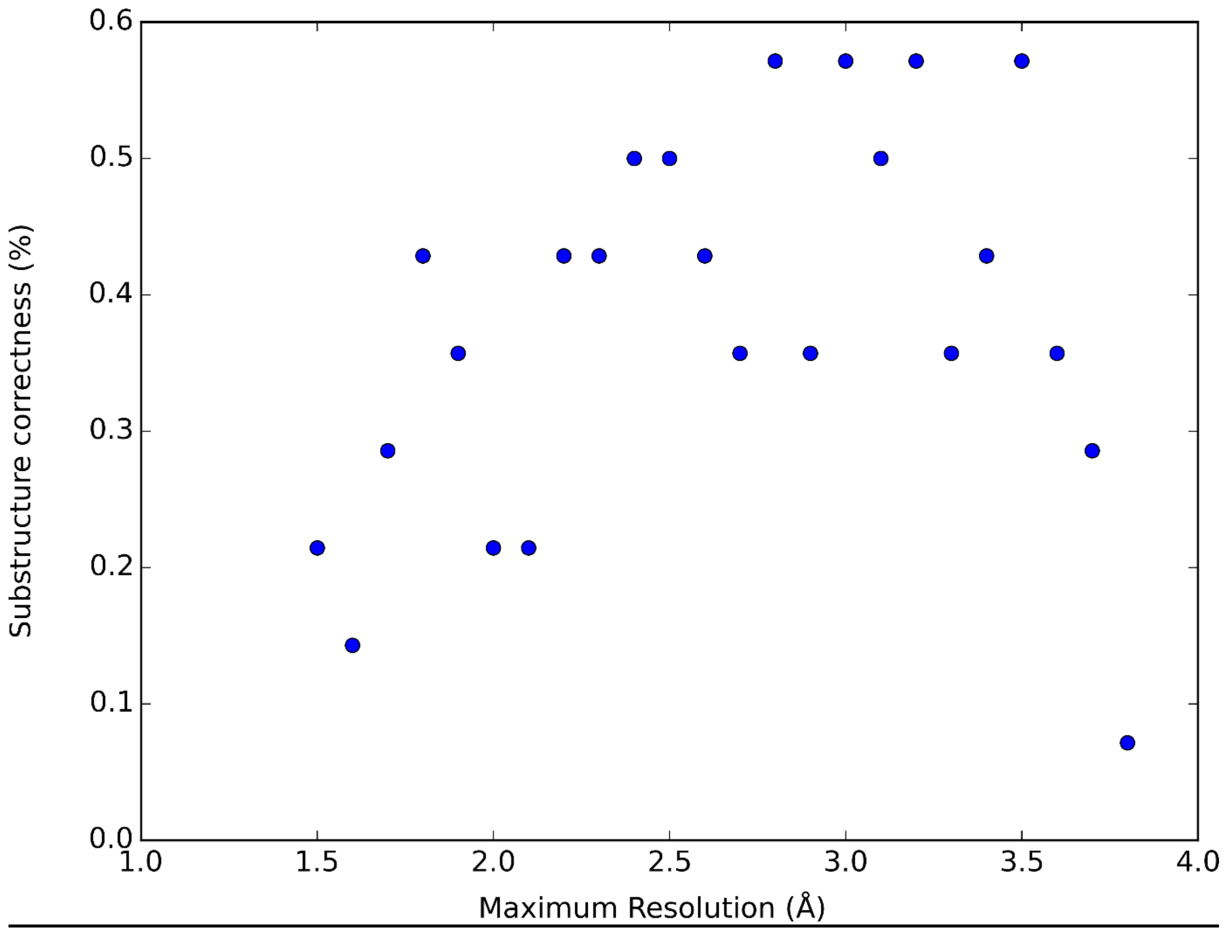

Figure S5 Substructure correctness compared to a known reference model as a function of the maximal resolution in SHELX (SHEL keyword).

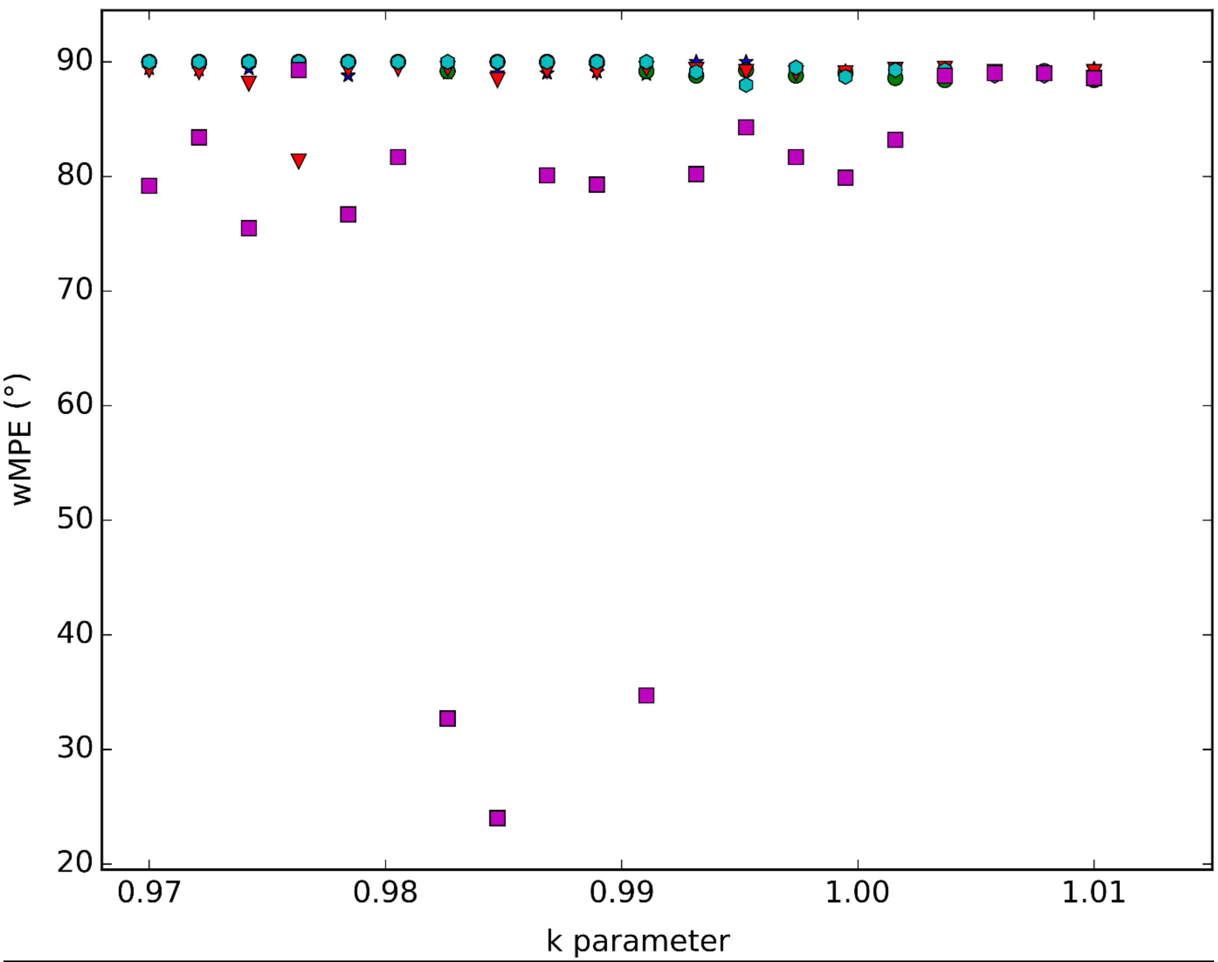

Figure S6 Phase errors of experimental phasing for different doses as a function of the K scaling factor. The wMPE is the best phase error compared to a refined model. Blue stars represent 1.16 MGy, green dots 1.74 MGy, red triangles 2.32 MGy, cyan hexagons 2.90 MGy and purple squares 3.46 MGy. Independently of the K scaling factor, only the highest dose allows for proper phase determination.
